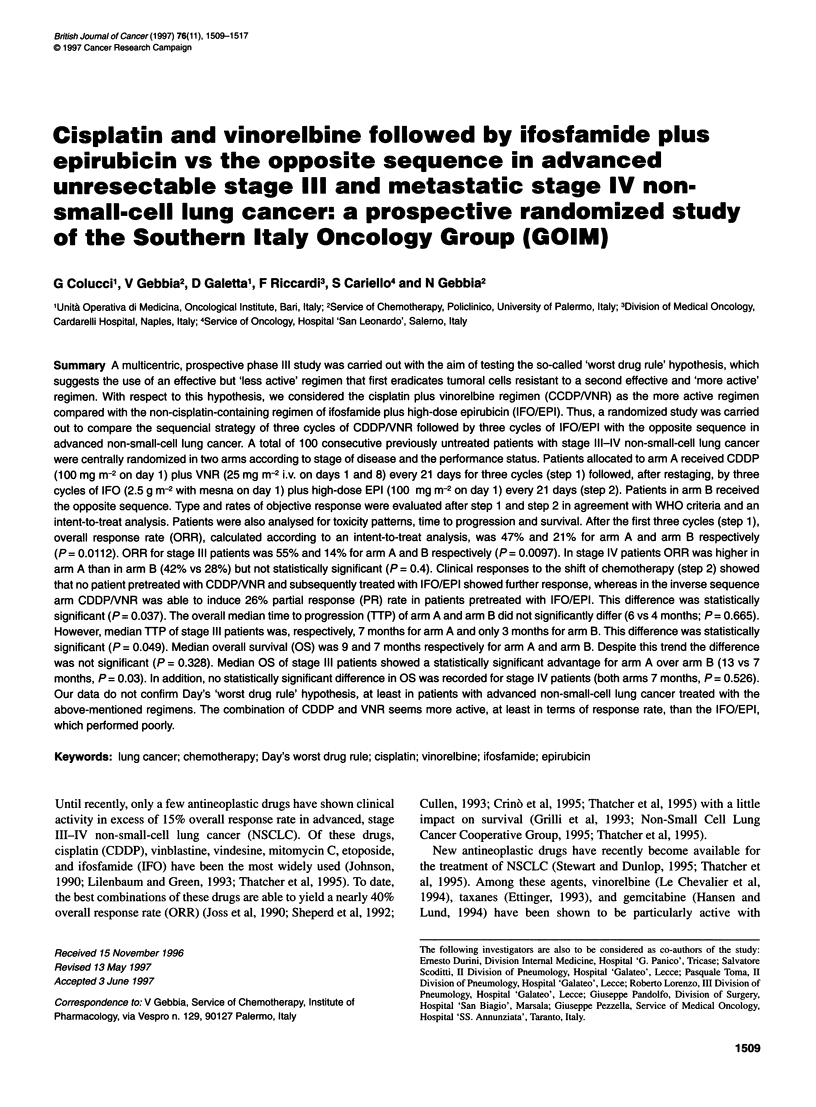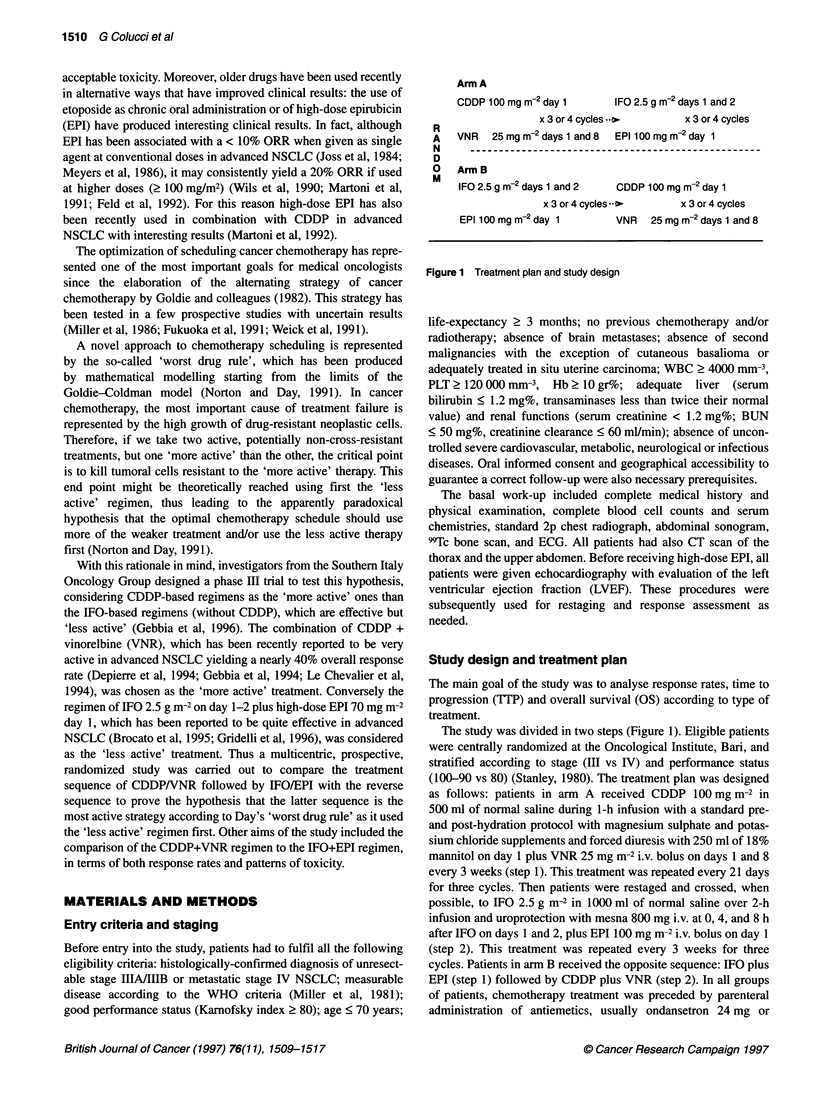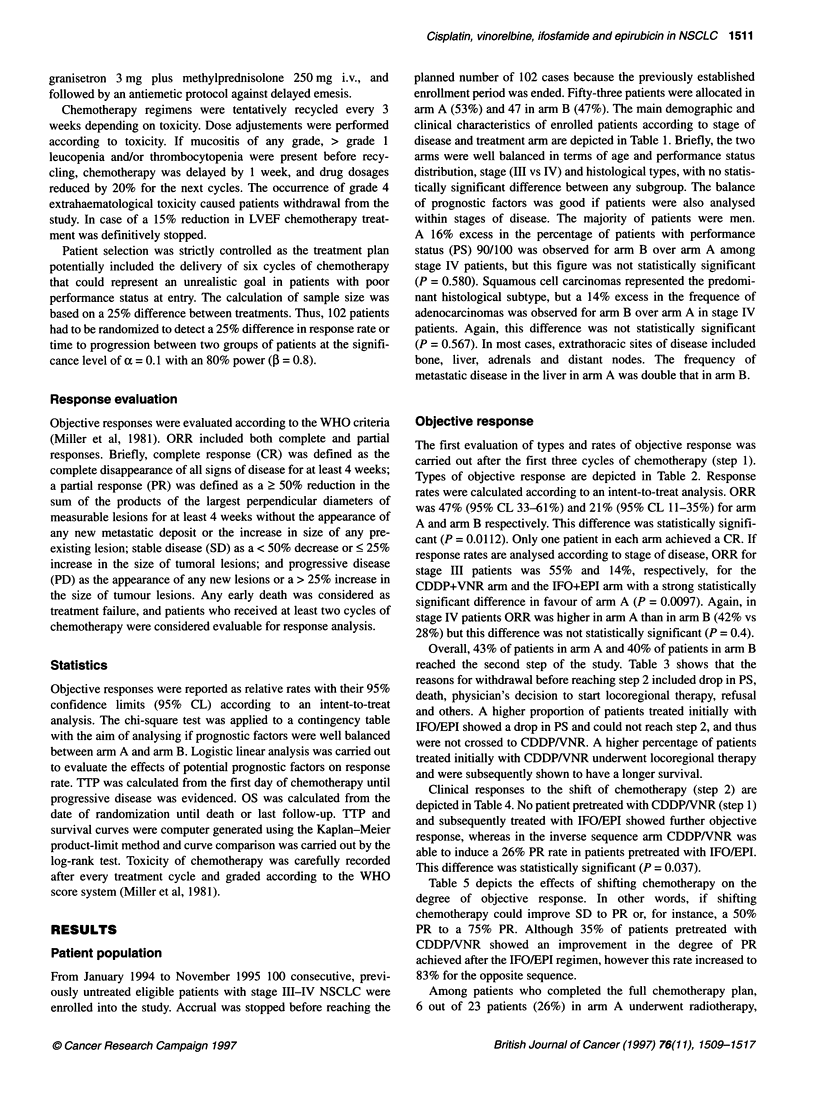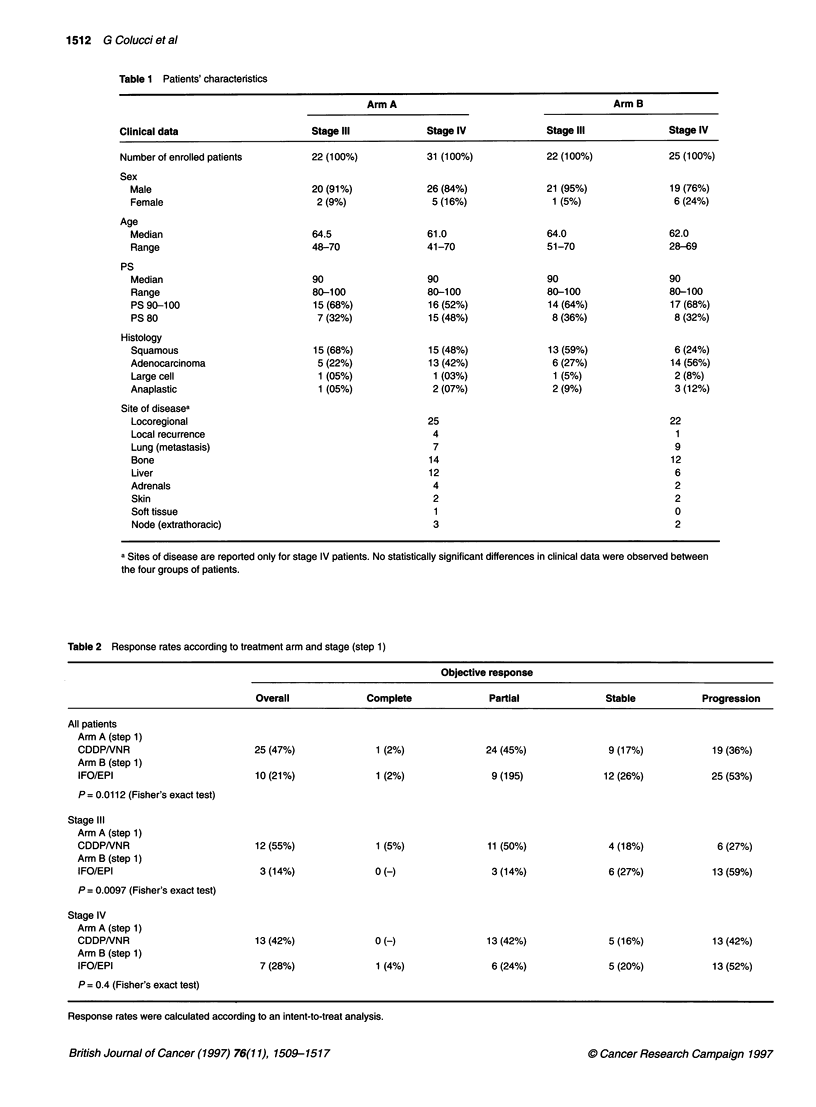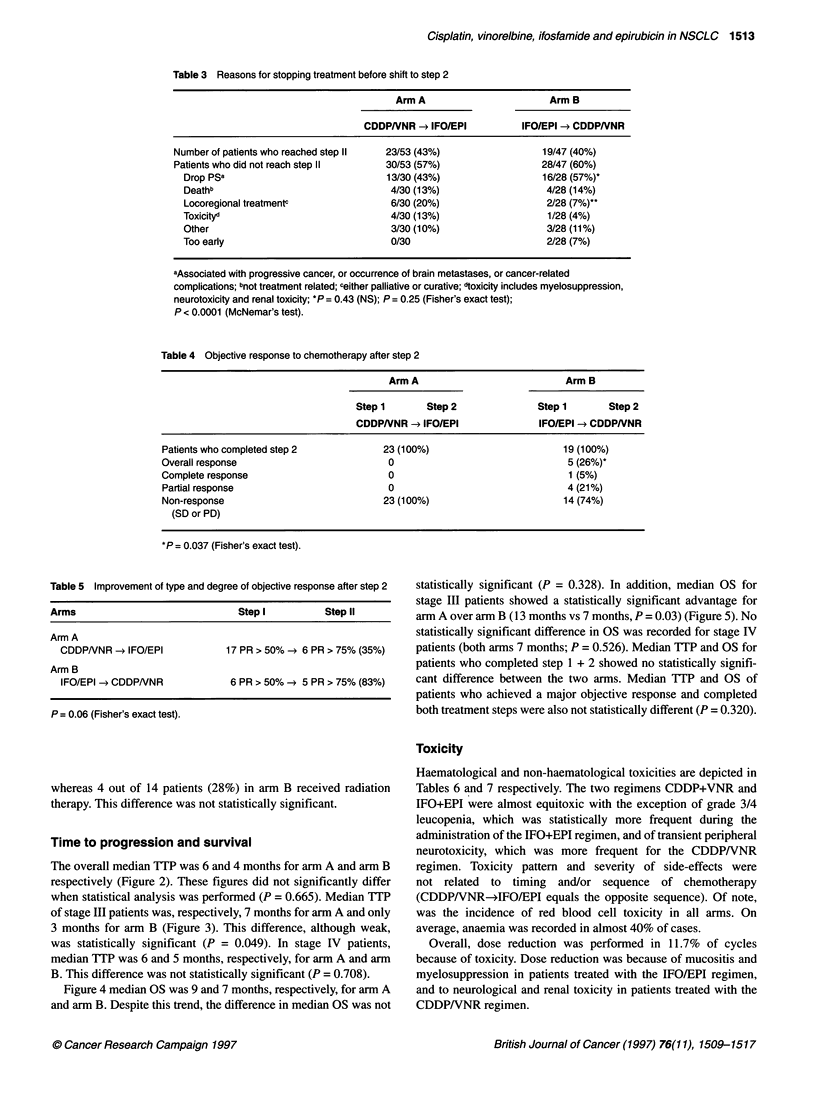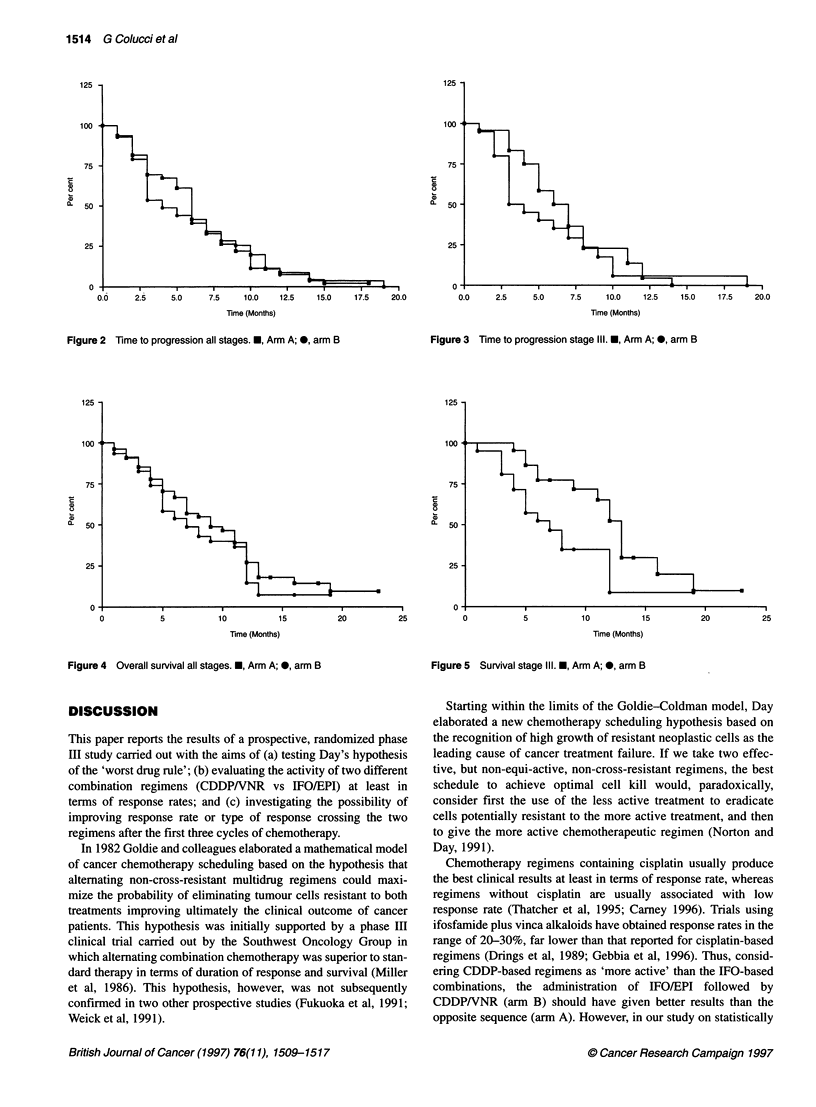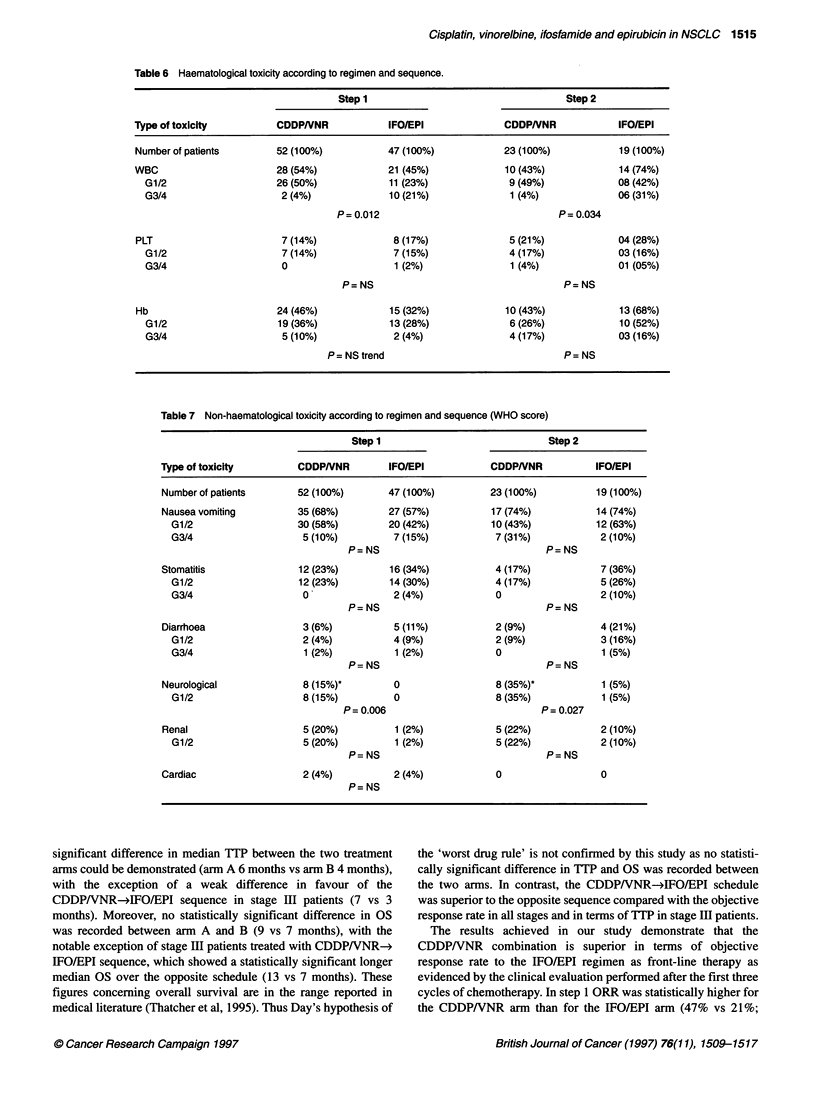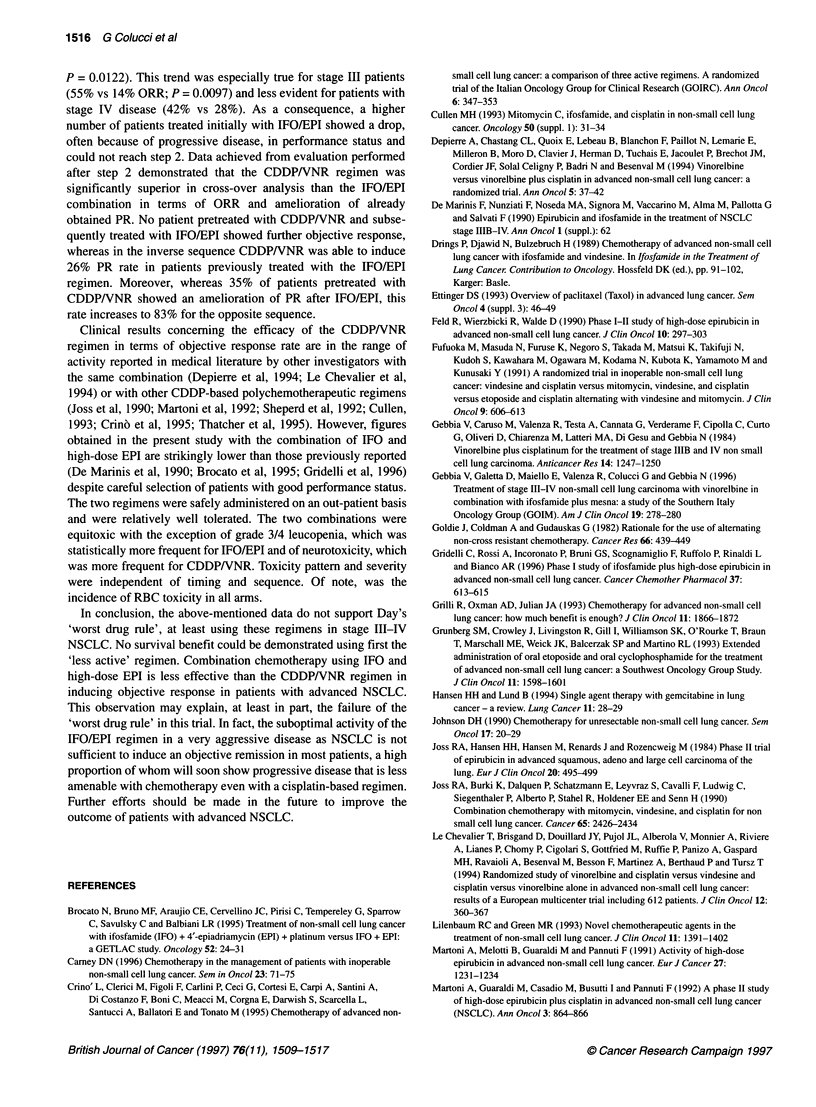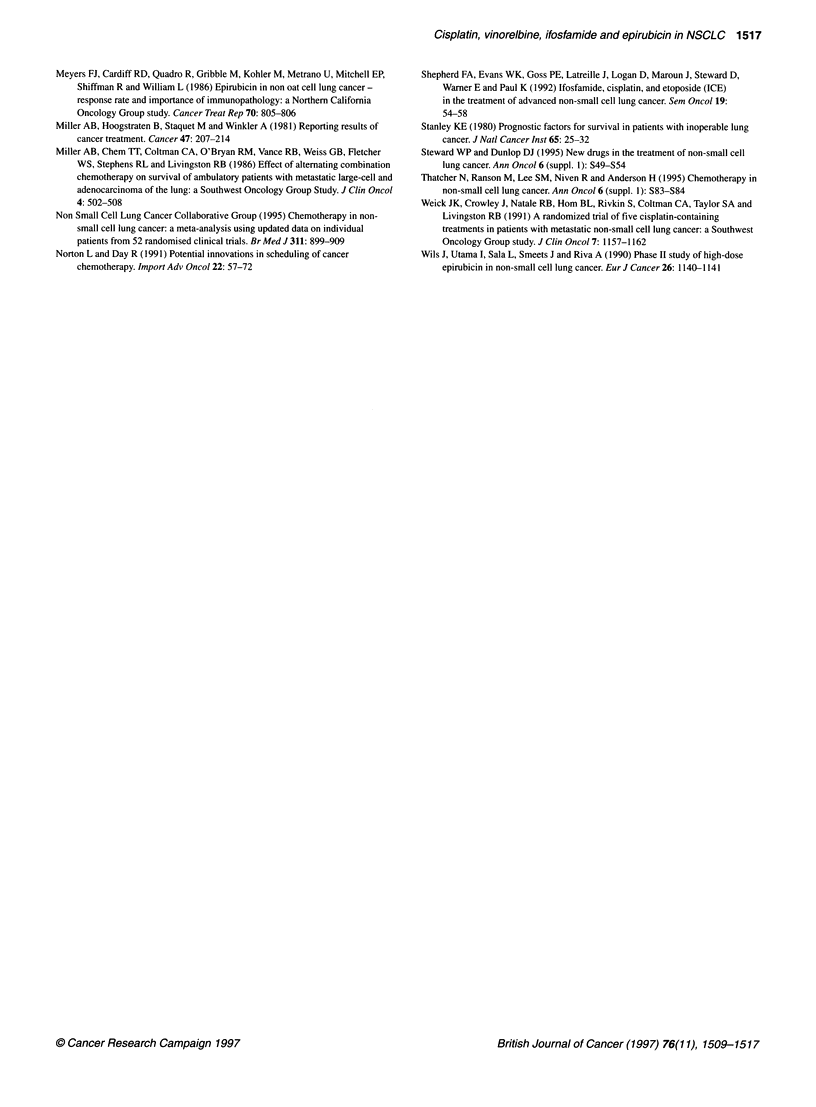# Cisplatin and vinorelbine followed by ifosfamide plus epirubicin vs the opposite sequence in advanced unresectable stage III and metastatic stage IV non-small-cell lung cancer: a prospective randomized study of the Southern Italy Oncology Group (GOIM).

**DOI:** 10.1038/bjc.1997.586

**Published:** 1997

**Authors:** G. Colucci, V. Gebbia, D. Galetta, F. Riccardi, S. Cariello, N. Gebbia

**Affiliations:** UnitÃ Operativa di Medicina, Oncological Institute, Bari, Italy.

## Abstract

A multicentric, prospective phase III study was carried out with the aim of testing the so-called 'worst drug rule' hypothesis, which suggests the use of an effective but 'less active' regimen that first eradicates tumoral cells resistant to a second effective and 'more active' regimen. With respect to this hypothesis, we considered the cisplatin plus vinorelbine regimen (CCDP/VNR) as the more active regimen compared with the non-cisplatin-containing regimen of ifosfamide plus high-dose epirubicin (IFO/EPI). Thus, a randomized study was carried out to compare the sequencial strategy of three cycles of CDDP/VNR followed by three cycles of IFO/EPI with the opposite sequence in advanced non-small-cell lung cancer. A total of 100 consecutive previously untreated patients with stage III-IV non-small-cell lung cancer were centrally randomized in two arms according to stage of disease and the performance status. Patients allocated to arm A received CDDP (100 mg m-2 on day 1) plus VNR (25 mg m-2 i.v. on days 1 and 8) every 21 days for three cycles (step 1) followed, after restaging, by three cycles of IFO (2.5 g m-2 with mesna on day 1) plus high-dose EPI (100 mg m-2 on day 1) every 21 days (step 2). Patients in arm B received the opposite sequence. Type and rates of objective response were evaluated after step 1 and step 2 in agreement with WHO criteria and an intent-to-treat analysis. Patients were also analysed for toxicity patterns, time to progression and survival. After the first three cycles (step 1), overall response rate (ORR), calculated according to an intent-to-treat analysis, was 47% and 21% for arm A and arm B respectively (P = 0.0112). ORR for stage III patients was 55% and 14% for arm A and B respectively (P = 0.0097). In stage IV patients ORR was higher in arm A than in arm B (42% vs 28%) but not statistically significant (P = 0.4). Clinical responses to the shift of chemotherapy (step 2) showed that no patient pretreated with CDDP/VNR and subsequently treated with IFO/EPI showed further response, whereas in the inverse sequence arm CDDP/VNR was able to induce 26% partial response (PR) rate in patients pretreated with IFO/EPI. This difference was statistically significant (P = 0.037). The overall median time to progression (TTP) of arm A and arm B did not significantly differ (6 vs 4 months; P = 0.665). However, median TTP of stage III patients was, respectively, 7 months for arm A and only 3 months for arm B. This difference was statistically significant (P = 0.049). Median overall survival (OS) was 9 and 7 months respectively for arm A and arm B. Despite this trend the difference was not significant (P = 0.328). Median OS of stage III patients showed a statistically significant advantage for arm A over arm B (13 vs 7 months, P = 0.03). In addition, no statistically significant difference in OS was recorded for stage IV patients (both arms 7 months, P = 0.526). Our data do not confirm Day's 'worst drug rule' hypothesis, at least in patients with advanced non-small-cell lung cancer treated with the above-mentioned regimens. The combination of CDDP and VNR seems more active, at least in terms of response rate, than the IFO/EPI, which performed poorly.


British Journal of Cancer (1997) 76(11), 1509–1517
© 1997 Cancer Research Campaign

### Cisplatin and vinorelbine followed by ifosfamide plus epirubicin vs the opposite sequence in advanced unresectable stage III and metastatic stage IV non-small-cell lung cancer: a prospective randomized study of the Southern Italy Oncology Group (GOIM)

G Colucci[1], V Gebbia[2], D Galetta[1], F Riccardi[3], S Cariello[4] and N Gebbia[2]

[1]Unità Operativa di Medicina, Oncological Institute, Bari, Italy; [2]Service of Chemotherapy, Policlinico, University of Palermo, Italy; [3]Division of Medical Oncology, Cardarelli Hospital, Naples, Italy; [4]Service of Oncology, Hospital 'San Leonardo', Salerno, Italy

**Summary** A multicentric, prospective phase III study was carried out with the aim of testing the so-called 'worst drug rule' hypothesis, which suggests the use of an effective but 'less active' regimen that first eradicates tumoral cells resistant to a second effective and 'more active' regimen. With respect to this hypothesis, we considered the cisplatin plus vinorelbine regimen (CCDP/VNR) as the more active regimen compared with the non-cisplatin-containing regimen of ifosfamide plus high-dose epirubicin (IFO/EPI). Thus, a randomized study was carried out to compare the sequencial strategy of three cycles of CDDP/VNR followed by three cycles of IFO/EPI with the opposite sequence in advanced non-small-cell lung cancer. A total of 100 consecutive previously untreated patients with stage III–IV non-small-cell lung cancer were centrally randomized in two arms according to stage of disease and the performance status. Patients allocated to arm A received CDDP (100 mg $m^{-2}$ on day 1) plus VNR (25 mg $m^{-2}$ i.v. on days 1 and 8) every 21 days for three cycles (step 1) followed, after restaging, by three cycles of IFO (2.5 g $m^{-2}$ with mesna on day 1) plus high-dose EPI (100 mg $m^{-2}$ on day 1) every 21 days (step 2). Patients in arm B received the opposite sequence. Type and rates of objective response were evaluated after step 1 and step 2 in agreement with WHO criteria and an intent-to-treat analysis. Patients were also analysed for toxicity patterns, time to progression and survival. After the first three cycles (step 1), overall response rate (ORR), calculated according to an intent-to-treat analysis, was 47% and 21% for arm A and arm B respectively ($P$ = 0.0112). ORR for stage III patients was 55% and 14% for arm A and B respectively ($P$ = 0.0097). In stage IV patients ORR was higher in arm A than in arm B (42% vs 28%) but not statistically significant ($P$ = 0.4). Clinical responses to the shift of chemotherapy (step 2) showed that no patient pretreated with CDDP/VNR and subsequently treated with IFO/EPI showed further response, whereas in the inverse sequence arm CDDP/VNR was able to induce 26% partial response (PR) rate in patients pretreated with IFO/EPI. This difference was statistically significant ($P$ = 0.037). The overall median time to progression (TTP) of arm A and arm B did not significantly differ (6 vs 4 months; $P$ = 0.665). However, median TTP of stage III patients was, respectively, 7 months for arm A and only 3 months for arm B. This difference was statistically significant ($P$ = 0.049). Median overall survival (OS) was 9 and 7 months respectively for arm A and arm B. Despite this trend the difference was not significant ($P$ = 0.328). Median OS of stage III patients showed a statistically significant advantage for arm A over arm B (13 vs 7 months, $P$ = 0.03). In addition, no statistically significant difference in OS was recorded for stage IV patients (both arms 7 months, $P$ = 0.526). Our data do not confirm Day's 'worst drug rule' hypothesis, at least in patients with advanced non-small-cell lung cancer treated with the above-mentioned regimens. The combination of CDDP and VNR seems more active, at least in terms of response rate, than the IFO/EPI, which performed poorly.

**Keywords:** lung cancer; chemotherapy; Day's worst drug rule; cisplatin; vinorelbine; ifosfamide; epirubicin

Until recently, only a few antineoplastic drugs have shown clinical activity in excess of 15% overall response rate in advanced, stage III–IV non-small-cell lung cancer (NSCLC). Of these drugs, cisplatin (CDDP), vinblastine, vindesine, mitomycin C, etoposide, and ifosfamide (IFO) have been the most widely used (Johnson, 1990; Lilenbaum and Green, 1993; Thatcher et al, 1995). To date, the best combinations of these drugs are able to yield a nearly 40% overall response rate (ORR) (Joss et al, 1990; Sheperd et al, 1992; Cullen, 1993; Crinò et al, 1995; Thatcher et al, 1995) with a little impact on survival (Grilli et al, 1993; Non-Small Cell Lung Cancer Cooperative Group, 1995; Thatcher et al, 1995).

New antineoplastic drugs have recently become available for the treatment of NSCLC (Stewart and Dunlop, 1995; Thatcher et al, 1995). Among these agents, vinorelbine (Le Chevalier et al, 1994), taxanes (Ettinger, 1993), and gemcitabine (Hansen and Lund, 1994) have been shown to be particularly active with

*Received 15 November 1996*

*Revised 13 May 1997*

*Accepted 3 June 1997*

*Correspondence to:* V Gebbia, Service of Chemotherapy, Institute of Pharmacology, via Vespro n. 129, 90127 Palermo, Italy

The following investigators are also to be considered as co-authors of the study: Ernesto Durini, Division Internal Medicine, Hospital 'G. Panico', Tricase; Salvatore Scoditti, II Division of Pneumology, Hospital 'Galateo', Lecce; Pasquale Toma, II Division of Pneumology, Hospital 'Galateo', Lecce; Roberto Lorenzo, III Division of Pneumology, Hospital 'Galateo', Lecce; Giuseppe Pandolfo, Division of Surgery, Hospital 'San Biagio', Marsala; Giuseppe Pezzella, Service of Medical Oncology, Hospital 'SS. Annunziata', Taranto, Italy.

acceptable toxicity. Moreover, older drugs have been used recently in alternative ways that have improved clinical results: the use of etoposide as chronic oral administration or of high-dose epirubicin (EPI) have produced interesting clinical results. In fact, although EPI has been associated with a < 10% ORR when given as single agent at conventional doses in advanced NSCLC (Joss et al, 1984; Meyers et al, 1986), it may consistently yield a 20% ORR if used at higher doses (≥ 100 mg/m²) (Wils et al, 1990; Martoni et al, 1991; Feld et al, 1992). For this reason high-dose EPI has also been recently used in combination with CDDP in advanced NSCLC with interesting results (Martoni et al, 1992).

The optimization of scheduling cancer chemotherapy has represented one of the most important goals for medical oncologists since the elaboration of the alternating strategy of cancer chemotherapy by Goldie and colleagues (1982). This strategy has been tested in a few prospective studies with uncertain results (Miller et al, 1986; Fukuoka et al, 1991; Weick et al, 1991).

A novel approach to chemotherapy scheduling is represented by the so-called 'worst drug rule', which has been produced by mathematical modelling starting from the limits of the Goldie–Coldman model (Norton and Day, 1991). In cancer chemotherapy, the most important cause of treatment failure is represented by the high growth of drug-resistant neoplastic cells. Therefore, if we take two active, potentially non-cross-resistant treatments, but one 'more active' than the other, the critical point is to kill tumoral cells resistant to the 'more active' therapy. This end point might be theoretically reached using first the 'less active' regimen, thus leading to the apparently paradoxical hypothesis that the optimal chemotherapy schedule should use more of the weaker treatment and/or use the less active therapy first (Norton and Day, 1991).

With this rationale in mind, investigators from the Southern Italy Oncology Group designed a phase III trial to test this hypothesis, considering CDDP-based regimens as the 'more active' ones than the IFO-based regimens (without CDDP), which are effective but 'less active' (Gebbia et al, 1996). The combination of CDDP + vinorelbine (VNR), which has been recently reported to be very active in advanced NSCLC yielding a nearly 40% overall response rate (Depierre et al, 1994; Gebbia et al, 1994; Le Chevalier et al, 1994), was chosen as the 'more active' treatment. Conversely the regimen of IFO 2.5 g m$^{-2}$ on day 1–2 plus high-dose EPI 70 mg m$^{-2}$ day 1, which has been reported to be quite effective in advanced NSCLC (Brocato et al, 1995; Gridelli et al, 1996), was considered as the 'less active' treatment. Thus a multicentric, prospective, randomized study was carried out to compare the treatment sequence of CDDP/VNR followed by IFO/EPI with the reverse sequence to prove the hypothesis that the latter sequence is the most active strategy according to Day's 'worst drug rule' as it used the 'less active' regimen first. Other aims of the study included the comparison of the CDDP+VNR regimen to the IFO+EPI regimen, in terms of both response rates and patterns of toxicity.

### MATERIALS AND METHODS

### Entry criteria and staging

Before entry into the study, patients had to fulfil all the following eligibility criteria: histologically-confirmed diagnosis of unresectable stage IIIA/IIIB or metastatic stage IV NSCLC; measurable disease according to the WHO criteria (Miller et al, 1981); good performance status (Karnofsky index ≥ 80); age ≤ 70 years;

| RANDOM | Step 1 | | Step 2 |
|---|---|---|---|
| **Arm A** | | | |
| | CDDP 100 mg m$^{-2}$ day 1 | x 3 or 4 cycles --> | IFO 2.5 g m$^{-2}$ days 1 and 2 (x 3 or 4 cycles) |
| | VNR 25 mg m$^{-2}$ days 1 and 8 | | EPI 100 mg m$^{-2}$ day 1 |
| **Arm B** | | | |
| | IFO 2.5 g m$^{-2}$ days 1 and 2 | x 3 or 4 cycles --> | CDDP 100 mg m$^{-2}$ day 1 (x 3 or 4 cycles) |
| | EPI 100 mg m$^{-2}$ day 1 | | VNR 25 mg m$^{-2}$ days 1 and 8 |

**Figure 1** Treatment plan and study design life-expectancy ≥ 3 months; no previous chemotherapy and/or radiotherapy; absence of brain metastases; absence of second malignancies with the exception of cutaneous basalioma or adequately treated in situ uterine carcinoma; WBC ≥ 4000 mm$^{-3}$, PLT ≥ 120 000 mm$^{-3}$, Hb ≥ 10 gr%; adequate liver (serum bilirubin ≤ 1.2 mg%, transaminases less than twice their normal value) and renal functions (serum creatinine < 1.2 mg%; BUN ≤ 50 mg%, creatinine clearance ≤ 60 ml/min); absence of uncontrolled severe cardiovascular, metabolic, neurological or infectious diseases. Oral informed consent and geographical accessibility to guarantee a correct follow-up were also necessary prerequisites.

The basal work-up included complete medical history and physical examination, complete blood cell counts and serum chemistries, standard 2p chest radiograph, abdominal sonogram, $^{99}$Tc bone scan, and ECG. All patients had also CT scan of the thorax and the upper abdomen. Before receiving high-dose EPI, all patients were given echocardiography with evaluation of the left ventricular ejection fraction (LVEF). These procedures were subsequently used for restaging and response assessment as needed.

### Study design and treatment plan

The main goal of the study was to analyse response rates, time to progression (TTP) and overall survival (OS) according to type of treatment.

The study was divided in two steps (Figure 1). Eligible patients were centrally randomized at the Oncological Institute, Bari, and stratified according to stage (III vs IV) and performance status (100–90 vs 80) (Stanley, 1980). The treatment plan was designed as follows: patients in arm A received CDDP 100 mg m$^{-2}$ in 500 ml of normal saline during 1-h infusion with a standard pre- and post-hydration protocol with magnesium sulphate and potassium chloride supplements and forced diuresis with 250 ml of 18% mannitol on day 1 plus VNR 25 mg m$^{-2}$ i.v. bolus on days 1 and 8 every 3 weeks (step 1). This treatment was repeated every 21 days for three cycles. Then patients were restaged and crossed, when possible, to IFO 2.5 g m$^{-2}$ in 1000 ml of normal saline over 2-h infusion and uroprotection with mesna 800 mg i.v. at 0, 4, and 8 h after IFO on days 1 and 2, plus EPI 100 mg m$^{-2}$ i.v. bolus on day 1 (step 2). This treatment was repeated every 3 weeks for three cycles. Patients in arm B received the opposite sequence: IFO plus EPI (step 1) followed by CDDP plus VNR (step 2). In all groups of patients, chemotherapy treatment was preceded by parenteral administration of antiemetics, usually ondansetron 24 mg or

 *© Cancer Research Campaign 1997*

granisetron 3 mg plus methylprednisolone 250 mg i.v., and followed by an antiemetic protocol against delayed emesis.

Chemotherapy regimens were tentatively recycled every 3 weeks depending on toxicity. Dose adjustements were performed according to toxicity. If mucositis of any grade, > grade 1 leucopenia and/or thrombocytopenia were present before recycling, chemotherapy was delayed by 1 week, and drug dosages reduced by 20% for the next cycles. The occurrence of grade 4 extrahaematological toxicity caused patients withdrawal from the study. In case of a 15% reduction in LVEF chemotherapy treatment was definitively stopped.

Patient selection was strictly controlled as the treatment plan potentially included the delivery of six cycles of chemotherapy that could represent an unrealistic goal in patients with poor performance status at entry. The calculation of sample size was based on a 25% difference between treatments. Thus, 102 patients had to be randomized to detect a 25% difference in response rate or time to progression between two groups of patients at the significance level of $\alpha = 0.1$ with an 80% power ($\beta = 0.8$).

### Response evaluation

Objective responses were evaluated according to the WHO criteria (Miller et al, 1981). ORR included both complete and partial responses. Briefly, complete response (CR) was defined as the complete disappearance of all signs of disease for at least 4 weeks; a partial response (PR) was defined as a ≥ 50% reduction in the sum of the products of the largest perpendicular diameters of measurable lesions for at least 4 weeks without the appearance of any new metastatic deposit or the increase in size of any pre-existing lesion; stable disease (SD) as a < 50% decrease or ≤ 25% increase in the size of tumoral lesions; and progressive disease (PD) as the appearance of any new lesions or a > 25% increase in the size of tumour lesions. Any early death was considered as treatment failure, and patients who received at least two cycles of chemotherapy were considered evaluable for response analysis.

### Statistics

Objective responses were reported as relative rates with their 95% confidence limits (95% CL) according to an intent-to-treat analysis. The chi-square test was applied to a contingency table with the aim of analysing if prognostic factors were well balanced between arm A and arm B. Logistic linear analysis was carried out to evaluate the effects of potential prognostic factors on response rate. TTP was calculated from the first day of chemotherapy until progressive disease was evidenced. OS was calculated from the date of randomization until death or last follow-up. TTP and survival curves were computer generated using the Kaplan–Meier product-limit method and curve comparison was carried out by the log-rank test. Toxicity of chemotherapy was carefully recorded after every treatment cycle and graded according to the WHO score system (Miller et al, 1981).

### RESULTS

### Patient population

From January 1994 to November 1995 100 consecutive, previously untreated eligible patients with stage III–IV NSCLC were enrolled into the study. Accrual was stopped before reaching the planned number of 102 cases because the previously established enrollment period was ended. Fifty-three patients were allocated in arm A (53%) and 47 in arm B (47%). The main demographic and clinical characteristics of enrolled patients according to stage of disease and treatment arm are depicted in Table 1. Briefly, the two arms were well balanced in terms of age and performance status distribution, stage (III vs IV) and histological types, with no statistically significant difference between any subgroup. The balance of prognostic factors was good if patients were also analysed within stages of disease. The majority of patients were men. A 16% excess in the percentage of patients with performance status (PS) 90/100 was observed for arm B over arm A among stage IV patients, but this figure was not statistically significant ($P = 0.580$). Squamous cell carcinomas represented the predominant histological subtype, but a 14% excess in the frequence of adenocarcinomas was observed for arm B over arm A in stage IV patients. Again, this difference was not statistically significant ($P = 0.567$). In most cases, extrathoracic sites of disease included bone, liver, adrenals and distant nodes. The frequency of metastatic disease in the liver in arm A was double that in arm B.

### Objective response

The first evaluation of types and rates of objective response was carried out after the first three cycles of chemotherapy (step 1). Types of objective response are depicted in Table 2. Response rates were calculated according to an intent-to-treat analysis. ORR was 47% (95% CL 33–61%) and 21% (95% CL 11–35%) for arm A and arm B respectively. This difference was statistically significant ($P = 0.0112$). Only one patient in each arm achieved a CR. If response rates are analysed according to stage of disease, ORR for stage III patients was 55% and 14%, respectively, for the CDDP+VNR arm and the IFO+EPI arm with a strong statistically significant difference in favour of arm A ($P = 0.0097$). Again, in stage IV patients ORR was higher in arm A than in arm B (42% vs 28%) but this difference was not statistically significant ($P = 0.4$).

Overall, 43% of patients in arm A and 40% of patients in arm B reached the second step of the study. Table 3 shows that the reasons for withdrawal before reaching step 2 included drop in PS, death, physician's decision to start locoregional therapy, refusal and others. A higher proportion of patients treated initially with IFO/EPI showed a drop in PS and could not reach step 2, and thus were not crossed to CDDP/VNR. A higher percentage of patients treated initially with CDDP/VNR underwent locoregional therapy and were subsequently shown to have a longer survival.

Clinical responses to the shift of chemotherapy (step 2) are depicted in Table 4. No patient pretreated with CDDP/VNR (step 1) and subsequently treated with IFO/EPI showed further objective response, whereas in the inverse sequence arm CDDP/VNR was able to induce a 26% PR rate in patients pretreated with IFO/EPI. This difference was statistically significant ($P = 0.037$).

Table 5 depicts the effects of shifting chemotherapy on the degree of objective response. In other words, if shifting chemotherapy could improve SD to PR or, for instance, a 50% PR to a 75% PR. Although 35% of patients pretreated with CDDP/VNR showed an improvement in the degree of PR achieved after the IFO/EPI regimen, however this rate increased to 83% for the opposite sequence.

Among patients who completed the full chemotherapy plan, 6 out of 23 patients (26%) in arm A underwent radiotherapy,

© Cancer Research Campaign 1997

**Table 1** Patients' characteristics

| Clinical data | Arm A Stage III | Arm A Stage IV | Arm B Stage III | Arm B Stage IV |
|---|---|---|---|---|
| Number of enrolled patients | 22 (100%) | 31 (100%) | 22 (100%) | 25 (100%) |
| Sex | | | | |
| Male | 20 (91%) | 26 (84%) | 21 (95%) | 19 (76%) |
| Female | 2 (9%) | 5 (16%) | 1 (5%) | 6 (24%) |
| Age | | | | |
| Median | 64.5 | 61.0 | 64.0 | 62.0 |
| Range | 48–70 | 41–70 | 51–70 | 28–69 |
| PS | | | | |
| Median | 90 | 90 | 90 | 90 |
| Range | 80–100 | 80–100 | 80–100 | 80–100 |
| PS 90–100 | 15 (68%) | 16 (52%) | 14 (64%) | 17 (68%) |
| PS 80 | 7 (32%) | 15 (48%) | 8 (36%) | 8 (32%) |
| Histology | | | | |
| Squamous | 15 (68%) | 15 (48%) | 13 (59%) | 6 (24%) |
| Adenocarcinoma | 5 (22%) | 13 (42%) | 6 (27%) | 14 (56%) |
| Large cell | 1 (05%) | 1 (03%) | 1 (5%) | 2 (8%) |
| Anaplastic | 1 (05%) | 2 (07%) | 2 (9%) | 3 (12%) |
| Site of disease[a] | | | | |
| Locoregional | | 25 | | 22 |
| Local recurrence | | 4 | | 1 |
| Lung (metastasis) | | 7 | | 9 |
| Bone | | 14 | | 12 |
| Liver | | 12 | | 6 |
| Adrenals | | 4 | | 2 |
| Skin | | 2 | | 2 |
| Soft tissue | | 1 | | 0 |
| Node (extrathoracic) | | 3 | | 2 |

[a] Sites of disease are reported only for stage IV patients. No statistically significant differences in clinical data were observed between the four groups of patients.

**Table 2** Response rates according to treatment arm and stage (step 1)

| | Objective response Overall | Complete | Partial | Stable | Progression |
|---|---|---|---|---|---|
| All patients | | | | | |
| Arm A (step 1) | | | | | |
| CDDP/VNR | 25 (47%) | 1 (2%) | 24 (45%) | 9 (17%) | 19 (36%) |
| Arm B (step 1) | | | | | |
| IFO/EPI | 10 (21%) | 1 (2%) | 9 (195) | 12 (26%) | 25 (53%) |
| $P = 0.0112$ (Fisher's exact test) | | | | | |
| Stage III | | | | | |
| Arm A (step 1) | | | | | |
| CDDP/VNR | 12 (55%) | 1 (5%) | 11 (50%) | 4 (18%) | 6 (27%) |
| Arm B (step 1) | | | | | |
| IFO/EPI | 3 (14%) | 0 (–) | 3 (14%) | 6 (27%) | 13 (59%) |
| $P = 0.0097$ (Fisher's exact test) | | | | | |
| Stage IV | | | | | |
| Arm A (step 1) | | | | | |
| CDDP/VNR | 13 (42%) | 0 (–) | 13 (42%) | 5 (16%) | 13 (42%) |
| Arm B (step 1) | | | | | |
| IFO/EPI | 7 (28%) | 1 (4%) | 6 (24%) | 5 (20%) | 13 (52%) |
| $P = 0.4$ (Fisher's exact test) | | | | | |

Response rates were calculated according to an intent-to-treat analysis.

 © Cancer Research Campaign 1997

**Table 3** Reasons for stopping treatment before shift to step 2

| | Arm A | Arm B |
|---|---|---|
| | CDDP/VNR → IFO/EPI | IFO/EPI → CDDP/VNR |
| Number of patients who reached step II | 23/53 (43%) | 19/47 (40%) |
| Patients who did not reach step II | 30/53 (57%) | 28/47 (60%) |
| Drop PS[a] | 13/30 (43%) | 16/28 (57%)* |
| Death[b] | 4/30 (13%) | 4/28 (14%) |
| Locoregional treatment[c] | 6/30 (20%) | 2/28 (7%)** |
| Toxicity[d] | 4/30 (13%) | 1/28 (4%) |
| Other | 3/30 (10%) | 3/28 (11%) |
| Too early | 0/30 | 2/28 (7%) |

[a]Associated with progressive cancer, or occurrence of brain metastases, or cancer-related complications; [b]not treatment related; [c]either palliative or curative; [d]toxicity includes myelosuppression, neurotoxicity and renal toxicity; $^*P = 0.43$ (NS); $P = 0.25$ (Fisher's exact test); $P < 0.0001$ (McNemar's test).

**Table 4** Objective response to chemotherapy after step 2

| | Arm A | Arm B |
|---|---|---|
| | Step 1 Step 2 | Step 1 Step 2 |
| | CDDP/VNR → IFO/EPI | IFO/EPI → CDDP/VNR |
| Patients who completed step 2 | 23 (100%) | 19 (100%) |
| Overall response | 0 | 5 (26%)* |
| Complete response | 0 | 1 (5%) |
| Partial response | 0 | 4 (21%) |
| Non-response (SD or PD) | 23 (100%) | 14 (74%) |

$^*P = 0.037$ (Fisher's exact test).

**Table 5** Improvement of type and degree of objective response after step 2

| Arms | Step I | Step II |
|---|---|---|
| Arm A CDDP/VNR → IFO/EPI | 17 PR > 50% → | 6 PR > 75% (35%) |
| Arm B IFO/EPI → CDDP/VNR | 6 PR > 50% → | 5 PR > 75% (83%) |

$P = 0.06$ (Fisher's exact test).

whereas 4 out of 14 patients (28%) in arm B received radiation therapy. This difference was not statistically significant.

### Time to progression and survival

The overall median TTP was 6 and 4 months for arm A and arm B respectively (Figure 2). These figures did not significantly differ when statistical analysis was performed ($P = 0.665$). Median TTP of stage III patients was, respectively, 7 months for arm A and only 3 months for arm B (Figure 3). This difference, although weak, was statistically significant ($P = 0.049$). In stage IV patients, median TTP was 6 and 5 months, respectively, for arm A and arm B. This difference was not statistically significant ($P = 0.708$).

Figure 4 median OS was 9 and 7 months, respectively, for arm A and arm B. Despite this trend, the difference in median OS was not statistically significant ($P = 0.328$). In addition, median OS for stage III patients showed a statistically significant advantage for arm A over arm B (13 months vs 7 months, $P = 0.03$) (Figure 5). No statistically significant difference in OS was recorded for stage IV patients (both arms 7 months; $P = 0.526$). Median TTP and OS for patients who completed step 1 + 2 showed no statistically significant difference between the two arms. Median TTP and OS of patients who achieved a major objective response and completed both treatment steps were also not statistically different ($P = 0.320$).

### Toxicity

Haematological and non-haematological toxicities are depicted in Tables 6 and 7 respectively. The two regimens CDDP+VNR and IFO+EPI were almost equitoxic with the exception of grade 3/4 leucopenia, which was statistically more frequent during the administration of the IFO+EPI regimen, and of transient peripheral neurotoxicity, which was more frequent for the CDDP/VNR regimen. Toxicity pattern and severity of side-effects were not related to timing and/or sequence of chemotherapy (CDDP/VNR→IFO/EPI equals the opposite sequence). Of note, was the incidence of red blood cell toxicity in all arms. On average, anaemia was recorded in almost 40% of cases.

Overall, dose reduction was performed in 11.7% of cycles because of toxicity. Dose reduction was because of mucositis and myelosuppression in patients treated with the IFO/EPI regimen, and to neurological and renal toxicity in patients treated with the CDDP/VNR regimen.

© Cancer Research Campaign 1997

**Figure 2** Time to progression all stages. ■, Arm A; ●, arm B

**Figure 3** Time to progression stage III. ■, Arm A; ●, arm B

**Figure 4** Overall survival all stages. ■, Arm A; ●, arm B

**Figure 5** Survival stage III. ■, Arm A; ●, arm B

### DISCUSSION

This paper reports the results of a prospective, randomized phase III study carried out with the aims of (a) testing Day's hypothesis of the 'worst drug rule'; (b) evaluating the activity of two different combination regimens (CDDP/VNR vs IFO/EPI) at least in terms of response rates; and (c) investigating the possibility of improving response rate or type of response crossing the two regimens after the first three cycles of chemotherapy.

In 1982 Goldie and colleagues elaborated a mathematical model of cancer chemotherapy scheduling based on the hypothesis that alternating non-cross-resistant multidrug regimens could maximize the probability of eliminating tumour cells resistant to both treatments improving ultimately the clinical outcome of cancer patients. This hypothesis was initially supported by a phase III clinical trial carried out by the Southwest Oncology Group in which alternating combination chemotherapy was superior to standard therapy in terms of duration of response and survival (Miller et al, 1986). This hypothesis, however, was not subsequently confirmed in two other prospective studies (Fukuoka et al, 1991; Weick et al, 1991).

Starting within the limits of the Goldie–Coldman model, Day elaborated a new chemotherapy scheduling hypothesis based on the recognition of high growth of resistant neoplastic cells as the leading cause of cancer treatment failure. If we take two effective, but non-equi-active, non-cross-resistant regimens, the best schedule to achieve optimal cell kill would, paradoxically, consider first the use of the less active treatment to eradicate cells potentially resistant to the more active treatment, and then to give the more active chemotherapeutic regimen (Norton and Day, 1991).

Chemotherapy regimens containing cisplatin usually produce the best clinical results at least in terms of response rate, whereas regimens without cisplatin are usually associated with low response rate (Thatcher et al, 1995; Carney 1996). Trials using ifosfamide plus vinca alkaloids have obtained response rates in the range of 20–30%, far lower than that reported for cisplatin-based regimens (Drings et al, 1989; Gebbia et al, 1996). Thus, considering CDDP-based regimens as 'more active' than the IFO-based combinations, the administration of IFO/EPI followed by CDDP/VNR (arm B) should have given better results than the opposite sequence (arm A). However, in our study on statistically

© Cancer Research Campaign 1997

**Table 6** Haematological toxicity according to regimen and sequence.

| Type of toxicity | Step 1 CDDP/VNR | Step 1 IFO/EPI | Step 2 CDDP/VNR | Step 2 IFO/EPI |
|---|---|---|---|---|
| Number of patients | 52 (100%) | 47 (100%) | 23 (100%) | 19 (100%) |
| WBC | 28 (54%) | 21 (45%) | 10 (43%) | 14 (74%) |
| G1/2 | 26 (50%) | 11 (23%) | 9 (49%) | 08 (42%) |
| G3/4 | 2 (4%) | 10 (21%) | 1 (4%) | 06 (31%) |
| | *P* = 0.012 | | *P* = 0.034 | |
| PLT | 7 (14%) | 8 (17%) | 5 (21%) | 04 (28%) |
| G1/2 | 7 (14%) | 7 (15%) | 4 (17%) | 03 (16%) |
| G3/4 | 0 | 1 (2%) | 1 (4%) | 01 (05%) |
| | *P* = NS | | *P* = NS | |
| Hb | 24 (46%) | 15 (32%) | 10 (43%) | 13 (68%) |
| G1/2 | 19 (36%) | 13 (28%) | 6 (26%) | 10 (52%) |
| G3/4 | 5 (10%) | 2 (4%) | 4 (17%) | 03 (16%) |
| | *P* = NS trend | | *P* = NS | |

**Table 7** Non-haematological toxicity according to regimen and sequence (WHO score)

| Type of toxicity | Step 1 CDDP/VNR | Step 1 IFO/EPI | Step 2 CDDP/VNR | Step 2 IFO/EPI |
|---|---|---|---|---|
| Number of patients | 52 (100%) | 47 (100%) | 23 (100%) | 19 (100%) |
| Nausea vomiting | 35 (68%) | 27 (57%) | 17 (74%) | 14 (74%) |
| G1/2 | 30 (58%) | 20 (42%) | 10 (43%) | 12 (63%) |
| G3/4 | 5 (10%) | 7 (15%) | 7 (31%) | 2 (10%) |
| | *P* = NS | | *P* = NS | |
| Stomatitis | 12 (23%) | 16 (34%) | 4 (17%) | 7 (36%) |
| G1/2 | 12 (23%) | 14 (30%) | 4 (17%) | 5 (26%) |
| G3/4 | 0 | 2 (4%) | 0 | 2 (10%) |
| | *P* = NS | | *P* = NS | |
| Diarrhoea | 3 (6%) | 5 (11%) | 2 (9%) | 4 (21%) |
| G1/2 | 2 (4%) | 4 (9%) | 2 (9%) | 3 (16%) |
| G3/4 | 1 (2%) | 1 (2%) | 0 | 1 (5%) |
| | *P* = NS | | *P* = NS | |
| Neurological | 8 (15%)* | 0 | 8 (35%)* | 1 (5%) |
| G1/2 | 8 (15%) | 0 | 8 (35%) | 1 (5%) |
| | *P* = 0.006 | | *P* = 0.027 | |
| Renal | 5 (20%) | 1 (2%) | 5 (22%) | 2 (10%) |
| G1/2 | 5 (20%) | 1 (2%) | 5 (22%) | 2 (10%) |
| | *P* = NS | | *P* = NS | |
| Cardiac | 2 (4%) | 2 (4%) | 0 | 0 |
| | *P* = NS | | | |

significant difference in median TTP between the two treatment arms could be demonstrated (arm A 6 months vs arm B 4 months), with the exception of a weak difference in favour of the CDDP/VNR→IFO/EPI sequence in stage III patients (7 vs 3 months). Moreover, no statistically significant difference in OS was recorded between arm A and B (9 vs 7 months), with the notable exception of stage III patients treated with CDDP/VNR→IFO/EPI sequence, which showed a statistically significant longer median OS over the opposite schedule (13 vs 7 months). These figures concerning overall survival are in the range reported in medical literature (Thatcher et al, 1995). Thus Day's hypothesis of the 'worst drug rule' is not confirmed by this study as no statistically significant difference in TTP and OS was recorded between the two arms. In contrast, the CDDP/VNR→IFO/EPI schedule was superior to the opposite sequence compared with the objective response rate in all stages and in terms of TTP in stage III patients.

The results achieved in our study demonstrate that the CDDP/VNR combination is superior in terms of objective response rate to the IFO/EPI regimen as front-line therapy as evidenced by the clinical evaluation performed after the first three cycles of chemotherapy. In step 1 ORR was statistically higher for the CDDP/VNR arm than for the IFO/EPI arm (47% vs 21%;

© Cancer Research Campaign 1997

P = 0.0122). This trend was especially true for stage III patients (55% vs 14% ORR; P = 0.0097) and less evident for patients with stage IV disease (42% vs 28%). As a consequence, a higher number of patients treated initially with IFO/EPI showed a drop, often because of progressive disease, in performance status and could not reach step 2. Data achieved from evaluation performed after step 2 demonstrated that the CDDP/VNR regimen was significantly superior in cross-over analysis than the IFO/EPI combination in terms of ORR and amelioration of already obtained PR. No patient pretreated with CDDP/VNR and subsequently treated with IFO/EPI showed further objective response, whereas in the inverse sequence CDDP/VNR was able to induce 26% PR rate in patients previously treated with the IFO/EPI regimen. Moreover, whereas 35% of patients pretreated with CDDP/VNR showed an amelioration of PR after IFO/EPI, this rate increases to 83% for the opposite sequence.

Clinical results concerning the efficacy of the CDDP/VNR regimen in terms of objective response rate are in the range of activity reported in medical literature by other investigators with the same combination (Depierre et al, 1994; Le Chevalier et al, 1994) or with other CDDP-based polychemotherapeutic regimens (Joss et al, 1990; Martoni et al, 1992; Sheperd et al, 1992; Cullen, 1993; Crinò et al, 1995; Thatcher et al, 1995). However, figures obtained in the present study with the combination of IFO and high-dose EPI are strikingly lower than those previously reported (De Marinis et al, 1990; Brocato et al, 1995; Gridelli et al, 1996) despite careful selection of patients with good performance status. The two regimens were safely administered on an out-patient basis and were relatively well tolerated. The two combinations were equitoxic with the exception of grade 3/4 leucopenia, which was statistically more frequent for IFO/EPI and of neurotoxicity, which was more frequent for CDDP/VNR. Toxicity pattern and severity were independent of timing and sequence. Of note, was the incidence of RBC toxicity in all arms.

In conclusion, the above-mentioned data do not support Day's 'worst drug rule', at least using these regimens in stage III–IV NSCLC. No survival benefit could be demonstrated using first the 'less active' regimen. Combination chemotherapy using IFO and high-dose EPI is less effective than the CDDP/VNR regimen in inducing objective response in patients with advanced NSCLC. This observation may explain, at least in part, the failure of the 'worst drug rule' in this trial. In fact, the suboptimal activity of the IFO/EPI regimen in a very aggressive disease as NSCLC is not sufficient to induce an objective remission in most patients, a high proportion of whom will soon show progressive disease that is less amenable with chemotherapy even with a cisplatin-based regimen. Further efforts should be made in the future to improve the outcome of patients with advanced NSCLC.

### REFERENCES

Brocato N, Bruno MF, Araujio CE, Cervellino JC, Pirisi C, Tempereley G, Sparrow C, Savulsky C and Balbiani LR (1995) Treatment of non-small cell lung cancer with ifosfamide (IFO) + 4′-epiadriamycin (EPI) + platinum versus IFO + EPI: a GETLAC study. *Oncology* **52**: 24–31

Carney DN (1996) Chemotherapy in the management of patients with inoperable non-small cell lung cancer. *Sem in Oncol* **23**: 71–75

Crino′ L, Clerici M, Figoli F, Carlini P, Ceci G, Cortesi E, Carpi A, Santini A, Di Costanzo F, Boni C, Meacci M, Corgna E, Darwish S, Scarcella L, Santucci A, Ballatori E and Tonato M (1995) Chemotherapy of advanced non-small cell lung cancer: a comparison of three active regimens. A randomized trial of the Italian Oncology Group for Clinical Research (GOIRC). *Ann Oncol* **6**: 347–353

Cullen MH (1993) Mitomycin C, ifosfamide, and cisplatin in non-small cell lung cancer. *Oncology* **50** (suppl. 1): 31–34

Depierre A, Chastang CL, Quoix E, Lebeau B, Blanchon F, Paillot N, Lemarie E, Milleron B, Moro D, Clavier J, Herman D, Tuchais E, Jacoulet P, Brechot JM, Cordier JF, Solal Celigny P, Badri N and Besenval M (1994) Vinorelbine versus vinorelbine plus cisplatin in advanced non-small cell lung cancer: a randomized trial. *Ann Oncol* **5**: 37–42

De Marinis F, Nunziati F, Noseda MA, Signora M, Vaccarino M, Alma M, Pallotta G and Salvati F (1990) Epirubicin and ifosfamide in the treatment of NSCLC stage IIIB–IV. *Ann Oncol* **1** (suppl.): 62

Drings P, Djawid N, Bulzebruch H (1989) Chemotherapy of advanced non-small cell lung cancer with ifosfamide and vindesine. In *Ifosfamide in the Treatment of Lung Cancer. Contribution to Oncology*. Hossfeld DK (ed.), pp. 91–102, Karger: Basle.

Ettinger DS (1993) Overview of paclitaxel (Taxol) in advanced lung cancer. *Sem Oncol* **4** (suppl. 3): 46–49

Feld R, Wierzbicki R, Walde D (1990) Phase I–II study of high-dose epirubicin in advanced non-small cell lung cancer. *J Clin Oncol* **10**: 297–303

Fufuoka M, Masuda N, Furuse K, Negoro S, Takada M, Matsui K, Takifuji N, Kudoh S, Kawahara M, Ogawara M, Kodama N, Kubota K, Yamamoto M and Kunusaki Y (1991) A randomized trial in inoperable non-small cell lung cancer: vindesine and cisplatin versus mitomycin, vindesine, and cisplatin versus etoposide and cisplatin alternating with vindesine and mitomycin. *J Clin Oncol* **9**: 606–613

Gebbia V, Caruso M, Valenza R, Testa A, Cannata G, Verderame F, Cipolla C, Curto G, Oliveri D, Chiarenza M, Latteri MA, Di Gesu and Gebbia N (1984) Vinorelbine plus cisplatinum for the treatment of stage IIIB and IV non small cell lung carcinoma. *Anticancer Res* **14**: 1247–1250

Gebbia V, Galetta D, Maiello E, Valenza R, Colucci G and Gebbia N (1996) Treatment of stage III–IV non-small cell lung carcinoma with vinorelbine in combination with ifosfamide plus mesna: a study of the Southern Italy Oncology Group (GOIM). *Am J Clin Oncol* **19**: 278–280

Goldie J, Coldman A and Gudauskas G (1982) Rationale for the use of alternating non-cross resistant chemotherapy. *Cancer Res* **66**: 439–449

Gridelli C, Rossi A, Incoronato P, Bruni GS, Scognamiglio F, Ruffolo P, Rinaldi L and Bianco AR (1996) Phase I study of ifosfamide plus high-dose epirubicin in advanced non-small cell lung cancer. *Cancer Chemother Pharmacol* **37**: 613–615

Grilli R, Oxman AD, Julian JA (1993) Chemotherapy for advanced non-small cell lung cancer: how much benefit is enough? *J Clin Oncol* **11**: 1866–1872

Grunberg SM, Crowley J, Livingston R, Gill I, Williamson SK, O'Rourke T, Braun T, Marschall ME, Weick JK, Balcerzak SP and Martino RL (1993) Extended administration of oral etoposide and oral cyclophosphamide for the treatment of advanced non-small cell lung cancer: a Southwest Oncology Group Study. *J Clin Oncol* **11**: 1598–1601

Hansen HH and Lund B (1994) Single agent therapy with gemcitabine in lung cancer – a review. *Lung Cancer* **11**: 28–29

Johnson DH (1990) Chemotherapy for unresectable non-small cell lung cancer. *Sem Oncol* **17**: 20–29

Joss RA, Hansen HH, Hansen M, Renards J and Rozencweig M (1984) Phase II trial of epirubicin in advanced squamous, adeno and large cell carcinoma of the lung. *Eur J Clin Oncol* **20**: 495–499

Joss RA, Burki K, Dalquen P, Schatzmann E, Leyvraz S, Cavalli F, Ludwig C, Siegenthaler P, Alberto P, Stahel R, Holdener EE and Senn H (1990) Combination chemotherapy with mitomycin, vindesine, and cisplatin for non small cell lung cancer. *Cancer* **65**: 2426–2434

Le Chevalier T, Brisgand D, Douillard JY, Pujol JL, Alberola V, Monnier A, Riviere A, Lianes P, Chomy P, Cigolari S, Gottfried M, Ruffie P, Panizo A, Gaspard MH, Ravaioli A, Besenval M, Besson F, Martinez A, Berthaud P and Tursz T (1994) Randomized study of vinorelbine and cisplatin versus vindesine and cisplatin versus vinorelbine alone in advanced non-small cell lung cancer: results of a European multicenter trial including 612 patients. *J Clin Oncol* **12**: 360–367

Lilenbaum RC and Green MR (1993) Novel chemotherapeutic agents in the treatment of non-small cell lung cancer. *J Clin Oncol* **11**: 1391–1402

Martoni A, Melotti B, Guaraldi M and Pannuti F (1991) Activity of high-dose epirubicin in advanced non-small cell lung cancer. *Eur J Cancer* **27**: 1231–1234

Martoni A, Guaraldi M, Casadio M, Busutti I and Pannuti F (1992) A phase II study of high-dose epirubicin plus cisplatin in advanced non-small cell lung cancer (NSCLC). *Ann Oncol* **3**: 864–866

© Cancer Research Campaign 1997

Meyers FJ, Cardiff RD, Quadro R, Gribble M, Kohler M, Metrano U, Mitchell EP, Shiffman R and William L (1986) Epirubicin in non oat cell lung cancer – response rate and importance of immunopathology: a Northern California Oncology Group study. *Cancer Treat Rep* **70**: 805–806

Miller AB, Hoogstraten B, Staquet M and Winkler A (1981) Reporting results of cancer treatment. *Cancer* **47**: 207–214

Miller AB, Chem TT, Coltman CA, O'Bryan RM, Vance RB, Weiss GB, Fletcher WS, Stephens RL and Livingston RB (1986) Effect of alternating combination chemotherapy on survival of ambulatory patients with metastatic large-cell and adenocarcinoma of the lung: a Southwest Oncology Group Study. *J Clin Oncol* **4**: 502–508

Non Small Cell Lung Cancer Collaborative Group (1995) Chemotherapy in non-small cell lung cancer: a meta-analysis using updated data on individual patients from 52 randomised clinical trials. *Br Med J* **311**: 899–909

Norton L and Day R (1991) Potential innovations in scheduling of cancer chemotherapy. *Import Adv Oncol* **22**: 57–72

Shepherd FA, Evans WK, Goss PE, Latreille J, Logan D, Maroun J, Steward D, Warner E and Paul K (1992) Ifosfamide, cisplatin, and etoposide (ICE) in the treatment of advanced non-small cell lung cancer. *Sem Oncol* **19**: 54–58

Stanley KE (1980) Prognostic factors for survival in patients with inoperable lung cancer. *J Natl Cancer Inst* **65**: 25–32

Steward WP and Dunlop DJ (1995) New drugs in the treatment of non-small cell lung cancer. *Ann Oncol* **6** (suppl. 1): S49–S54

Thatcher N, Ranson M, Lee SM, Niven R and Anderson H (1995) Chemotherapy in non-small cell lung cancer. *Ann Oncol* **6** (suppl. 1): S83–S84

Weick JK, Crowley J, Natale RB, Hom BL, Rivkin S, Coltman CA, Taylor SA and Livingston RB (1991) A randomized trial of five cisplatin-containing treatments in patients with metastatic non-small cell lung cancer: a Southwest Oncology Group study. *J Clin Oncol* **7**: 1157–1162

Wils J, Utama I, Sala L, Smeets J and Riva A (1990) Phase II study of high-dose epirubicin in non-small cell lung cancer. *Eur J Cancer* **26**: 1140–1141

© Cancer Research Campaign 1997